# N-Cofilin Can Compensate for the Loss of ADF in Excitatory Synapses

**DOI:** 10.1371/journal.pone.0026789

**Published:** 2011-10-28

**Authors:** Andreas Görlich, Michael Wolf, Anika-Maria Zimmermann, Christine B. Gurniak, Mumna Al Banchaabouchi, Marco Sassoè-Pognetto, Walter Witke, Eckhard Friauf, Marco B. Rust

**Affiliations:** 1 Neurobiology/Neurophysiology Group, University of Kaiserslautern, Kaiserslautern, Germany; 2 Institute of Genetics, University of Bonn, Bonn, Germany; 3 Mouse Biology Unit, European Mouse Biology Laboratory, Monterotondo, Italy; 4 Department of Anatomy, Pharmacology and Forensic Medicine and National Institute of Neuroscience-Italy, University of Turin, Turin, Italy; 5 Animal Physiology Group, University of Kaiserslautern, Kaiserslautern, Germany; University of Houston, United States of America

## Abstract

Actin plays important roles in a number of synaptic processes, including synaptic vesicle organization and exocytosis, mobility of postsynaptic receptors, and synaptic plasticity. However, little is known about the mechanisms that control actin at synapses. Actin dynamics crucially depend on LIM kinase 1 (LIMK1) that controls the activity of the actin depolymerizing proteins of the ADF/cofilin family. While analyses of mouse mutants revealed the importance of LIMK1 for both pre- and postsynaptic mechanisms, the ADF/cofilin family member n-cofilin appears to be relevant merely for postsynaptic plasticity, and not for presynaptic physiology. By means of immunogold electron microscopy and immunocytochemistry, we here demonstrate the presence of ADF (actin depolymerizing factor), a close homolog of n-cofilin, in excitatory synapses, where it is particularly enriched in presynaptic terminals. Surprisingly, genetic ablation of ADF in mice had no adverse effects on synapse structure or density as assessed by electron microscopy and by the morphological analysis of Golgi-stained hippocampal pyramidal cells. Moreover, a series of electrophysiological recordings in acute hippocampal slices revealed that presynaptic recruitment and exocytosis of synaptic vesicles as well as postsynaptic plasticity were unchanged in ADF mutant mice. The lack of synaptic defects may be explained by the elevated n-cofilin levels observed in synaptic structures of ADF mutants. Indeed, synaptic actin regulation was impaired in compound mutants lacking both ADF and n-cofilin, but not in ADF single mutants. From our results we conclude that n-cofilin can compensate for the loss of ADF in excitatory synapses. Further, our data suggest that ADF and n-cofilin cooperate in controlling synaptic actin content.

## Introduction

Actin is the most prominent protein at synapses and abundant in presynaptic terminals as well as postsynaptic spines [1]–[3]. Actin has been implicated in the organization, mobility, and exocytosis of synaptic vesicles (for review: [4]). It regulates the mobility of membrane proteins, such as neurotransmitter receptors [5]–[7], and promotes anchoring of receptors via its coupling to scaffolding proteins of the postsynaptic density (PSD) [8]. Moreover, actin regulation is essential for activity-dependent morphological changes of dendritic spines. Thereby actin is linked to synaptic plasticity, learning, and memory (for reviews: [4], [9]). Whereas the importance of actin for various synaptic processes is well accepted, little is known about the mechanisms that control actin at synapses.

Actin dynamics critically depend on the activity of ADF/cofilin that bind co-operatively to filamentous actin (F-actin), accelerate the dissociation of actin subunits and sever F-actin (for review: [10]). A tight regulation of ADF/cofilin activity is crucial for synaptic plasticity as shrinkage of dendritic spines requires active ADF/cofilin [11], while spine enlargement and long-term potentiation (LTP) depend on the inactivation of ADF/cofilin [7], [12]. A confined number of phosphatases and kinases, including LIM domain-containing serine/threonine kinases (LIM kinases), control ADF/cofilin activity via dephosphorylation (activation) and phosphorylation (inactivation) of a conserved serine residue at position 3 [13]. Dysregulation of the LIM kinase 1 (LIMK1)-ADF/cofilin pathway in humans is thought to contribute to the synaptic defects found in Williams' syndrome, a particular form of mental retardation, and autism spectrum disorders [14]. Indeed, genetic ablation of LIMK1 decreased ADF/cofilin phosphorylation in the mouse brain and affects synaptic vesicle exocytosis, neurotransmitter release, spine morphology, postsynaptic plasticity, learning, and memory [15]. Notably, inactivation of the ADF/cofilin family member n-cofilin (non-muscle cofilin) disturbed spine morphology and postsynaptic plasticity, while presynaptic physiology was fully preserved [6]. Thus, the molecule(s) acting downstream of LIMK1 in presynaptic terminals remain(s) unknown.

Besides n-cofilin, also its close homolog ADF (actin depolymerizing factor) is present in the adult mouse brain [6], [16], [17]. In the present study, we aimed to i) describe the subcellular localization of ADF in neurons and to ii) investigate whether ADF is relevant for synaptic structure and function. We demonstrate that ADF is present in pre- and postsynaptic compartments of excitatory synapses. Although ADF was particularly enriched in presynaptic axon terminals, presynaptic physiology was unchanged in ADF mutants. Likewise, ablation of ADF did not affect spine morphology, synaptic plasticity, learning and memory. Interestingly, we found elevated n-cofilin levels in synaptic structures of ADF mutant mice, suggesting that the loss of ADF may be compensated by n-cofilin. Indeed, compared to single mutant mice, synaptic actin levels were significantly increased in double mutants that lack both ADF and n-cofilin. Thus, our results revealed that n-cofilin has the capacity to compensate for the loss of ADF in synapses. Moreover, they let us to suggest that ADF together with n-cofilin controls the actin content in synapses.

## Methods

### Ethics Statement

Treatment of mice was in accordance with the German law for conducting animal experiments and followed the NIH guide for the care and use of laboratory animals. Killing of mice for tissue analysis as well as behavioral experiments were approved by the Landesuntersuchungsamt Rheinland-Pfalz (23 177-07/G09-2-001), mouse husbandry and breeding was approved by the City of Kaiserslautern – Referat Umweltschutz.

### Animals

Generation of ADF null mutants (ADF^−/−^) was described before [17]. Experiments were performed on ADF^−/−^ mice (ADF-KO). Age-matched wild-type littermates were used as controls (CTR).

### Biochemistry

#### Protein lysates

Tissue extracts from brain were prepared by homogenizing fresh tissue in ice-cold lysis buffer (in mM): 20 Tris-HCl (pH 8.0), 100 NaCl, 5 EGTA, 2 EDTA, supplemented with 0.5% TritonX-100 and EDTA-free complete protease inhibitor mix (Roche) using a tightly fitting douncer. Cytosolic and membranous protein fractions for determination of individual actin levels were separated by a centrifugation-based microsomal fractionation [18].

*Preparation of hippocampal synaptosomes* was essentially performed as described before [6]. Briefly, tissue was homogenized in homogenization solution containing (pH 7.4, in mM): 320 sucrose, 1 EDTA, 5 HEPES, supplemented with 0.1% bovine serum albumin and EDTA-free complete protease inhibitor mix (Roche) using a tight fitting dounce homogenizer. After removing nuclei and cell debris by a centrifugation step at 1,000 g for 5 min, supernatant was pelleted by centrifugation at 14,000 g for 10 min. The pellet containing synaptosomes was resuspended in Krebs-Ringer solution containing (pH 7.4, in mM): 140 NaCl, 5 KCl, 1 EDTA, 10 HEPES and 5 glucose. Synaptosomes were enriched on a floatation gradient consisting of 35% Percoll. *Primary antibodies* used for immunoblots were mouse anti-actin (1∶500; Abcam), rabbit anti-ADF (1∶5,000; Sigma Aldrich), mouse anti-PSD-95 (1∶1,000; Millipore), rabbit anti-synaptophysin (1∶1,000; Synaptic Systems), mouse anti-ß tubulin (1∶1,000; Sigma Aldrich). Generation of the rabbit anti-n-cofilin antibody was described before [17]. Primary antibodies were detected using horseradish peroxidase-conjugated goat anti-mouse and goat anti-rabbit IgG-antibodies (1∶5,000; Thermo Fisher Scientific) and Western Lightning Plus chemiluminescence detection kit (Perkin-Elmer).

### Morphology

#### Preembedding electron microscopy

Two mice were perfused with 2% paraformaldehyde and 0.1% glutaraldehyde in sodium acetate buffer, pH 6, for 1 min followed by 5 min perfusion with 2% formaldehyde and 0.1% glutaraldehyde in 0.1 M borate buffer, pH 9. Brains were postfixed overnight in the same fixative solution. 80 µm coronal sections were cut with a Vibratome. The sections were cryoprotected with 30% sucrose and frozen and thawed three times in liquid nitrogen to enhance the antibody penetration. Sections were blocked in 10% normal goat serum (NGS) in Tris-buffered saline (TBS, pH 7.4) and incubated with the primary antibody (anti-ADF; 1∶400; Sigma) diluted in TBS. The sections were then incubated with FluoroNanogold secondary antibodies (1∶200; Nanoprobes) and postfixed in 1% glutaraldehyde for 10 min. Labeled sections were incubated in GoldEnhance EM (Nanoprobes) for 8 min. Afterwards they were postfixed with 1% OsO4 in 0.1 M cacodylate buffer, dehydrated and embedded into Epon 812 (Serva). Serial thin sections were cut in CA1 and CA3 *stratum radiatum*, collected on single slot grids filmed with a pioloform solution and counterstained with uranyl acetate and lead citrate. They were observed and photographed in a JEM-1010 transmission electron microscope (Jeol) equipped with a side-mounted CCD camera (Mega View III; Soft Imaging System). Pictures were captured at a magnification of 40,000×. Gold particles were counted in at least 19 asymmetric synapses. *Golgi staining:* Brains of 6–7 weeks old mice were Golgi-stained using FD Rapid GolgiStain™ kit (FD Neurotechniques). Tissue impregnation and tissue section staining were performed according to the manufacturer's data sheet. Briefly, mice were perfused with 4% formaldehyde and brains were quickly removed from the skull and postfixed in 4% formaldehyde overnight. After incubation in impregnation solution and solution C, brains were imbedded in gelatin-albumin and cut into 100 µm coronal sections using a vibrating microtome (Campden Instruments Ltd.). Sections were mounted onto gelatinized glass slides, further processed for Golgi staining procedure, and finally mounted in Entellan (Merck). High magnification images of 2^nd^ order dendritic branches in the hippocampal CA1 *stratum radiatum* were generated by using an Axioskop microscope and a Plan-Neofluar 100×/1.30 oil immersion objective (Carl Zeiss). Spine density was measured using ImageJ 1.42q imaging software (NIH). Image acquisition and morphometric analysis were performed by an experimenter blind to the genotype of the mice. *Dendritic analysis:* Golgi-stained CA1 pyramidal neurons were manually traced on a transparent sheet by focusing through the depth of the slice using an Axioskop microscope and a Plan-Neofluar 20×/0.5 objective (Carl Zeiss). Only cells with minimal overlap of dendrites and without truncated dendrites were selected. For Sholl analysis, numbers of intersections of dendrites with concentric spheres centered on the soma with 20 µm (basal dendrites) or 40 µm (apical dendrites) steps were determined [19]. Tracing and Sholl analysis were done by an experimenter blind to the genotype of the mice.

#### Electron microscopy

6–7 week old mice were perfused with 1% formaldehyde/1% glutaraldehyde in phosphate buffer (0.1 M PB, pH 7.4). The brains were postfixed in the same fixative overnight, and small specimens taken from the dorsal hippocampus were postfixed in 1% OsO4 in 0.1 M cacodylate buffer, dehydrated, and embedded in epoxy resin. Ultrathin sections were stained with uranyl acetate and lead citrate and observed in a JEM-1010 transmission electron microscope. Spine density was assessed by analyzing 140 digitized images from three mice of each group. Images (30,000× magnification) were captured in the proximal part of CA1 *stratum radiatum*. Morphometric analyses were done on electron micrographs taken at 75,000× using ImageJ software. Synaptic structures were identified by presynaptic terminals with at least three synaptic vesicles, a visible synaptic cleft and a well-defined postsynaptic density. Image acquisition and morphometric analyses were performed by an experimenter blind to the genotype of the mice.

### Immunocytochemistry

#### Cell culture

Hippocampal cultures were prepared from newborn wildtype mice essentially as described before [20]. In brief, hippocampi were dissected in ice-cold Hank's Buffered Salt Solution (Invitrogen), incubated with trypsin (Invitrogen) for 15 min at 37°C and mechanically dissociated with fire polished Pasteur pipettes. Neurons (200 cells/mm^2^) were cultured in Neurobasal medium (Invitrogen) supplemented with 2% B27 (Invitrogen) and 2 mM glutamine (Invitrogen) on glass coverslips, coated with poly-L-lysine (Sigma Aldrich).

#### Immunocytochemistry and imaging

After 21 days in culture, cells were washed in phosphate buffered saline (PBS) and fixed in 4% paraformaldehyde/PBS for 15 min. Cells were permeabilized and blocked in blocking solution containing 10% goat serum (Invitrogen) and 0.2% Triton X-100 (Roth) in PBS for 1 h and subsequently washed with PBS. After washing, cells were incubated with primary antibodies for 0.5 to 2 h at 4°C in blocking solution. The following antibodies were used for immunocytochemistry: rabbit anti-ADF (1∶2,000; Sigma Aldrich), mouse anti-PSD-95 (1∶1,000; Millipore) and mouse anti-synaptophysin (1∶1,000; Synaptic Systems). After incubation with primary antibodies, cells were repeatedly washed in PBS and incubated with fluorescent secondary antibodies conjugated with Alexa Fluor-488 (1∶1,000; Invitrogen) and Alexa Fluor-546 (1∶1,000; Invitrogen) in carrier solution containing 5% goat serum and 0.2% Triton X-100 in PBS for 1 h at room temperature. For visualization of F-actin, cells were stained with rhodamine-phalloidin (1∶1000, Invitrogen) in carrier solution for 1 h at room temperature. Imaging of neurons was performed on a LSM700 confocal microscope (Zeiss) with a 20×/0.5 and a 63×/1.4 objective (Zeiss). For co-localization analysis, images were converted to binary scale with ImageJ 1.34 s software (NIH) and the relative numbers of spines or synaptic marker punctae (synaptophysin, PSD-95) co-localized with ADF were calculated.

### Electrophysiology

#### Tissue preparation

4–6 week old mice were sacrificed by cervical dislocation and their brains were rapidly removed and dissected in chilled solution (4°C) containing (in mM): 87 NaCl, 2 KCl, 0.5 CaCl_2_, 7 MgCl_2_, 26 NaHCO_3_, 1.25 NaH_2_PO_4_, 25 glucose, 75 sucrose, bubbled with a mixture of 95% O_2_/5% CO_2_, leading to a pH of 7.4. Horizontal hippocampal slices (thickness 300–370 µm) were cut with a VT1200S vibratome (Leica), preincubated for 30 min at 37°C and then transferred to the recording solution containing (in mM): 125 NaCl, 2.5 KCl, 4 CaCl_2_, 4 MgSO_4_, 26 NaHCO_3_, 1.25 NaH_2_PO_4_, 10 glucose, 2 sodium pyruvate, 3 myo-inositol, 0.44 ascorbic acid, bubbled with a mixture of 95% O_2_/5% CO_2_. Slices rested in this recording solution for at least one hour before recordings began.

#### Single cell recordings

Patch pipettes had resistances of 4–8 MΩ when filled with a solution containing (in mM): 117.5 CsMeSO_4_, 2.5 CsCl, 8 NaCl, 10 HEPES, 10 TEA, 0.2 EGTA, 4 Na_2_ATP, 0.6 Na_2_GTP, 5 QX-314 (pH adjusted to 7.2 with CsOH). Slices were transferred to the recording chamber, which was continuously perfused at a rate of 1.5–2 ml/min with recording solution at room temperature. CA1 hippocampal neurons were visualized with DIC-infrared optics using a 60×/1.0 water immersion objective on an upright Eclipe E600-FN microscope (Nikon). Electrophysiological responses were recorded with an EPC 10 patch-clamp amplifier and PatchMaster and FitMaster software (HEKA Elektronik). During recordings of miniature EPSCs (mEPSCs), 0.5 µM tetrodotoxin (TTX; Ascent Scientific), 100 µM picrotoxin (Ascent Scientific), and 250 µM trichlormethiazide (TCM; Sigma-Aldrich) were washed in. CA1 hippocampal neurons were voltage clamped at −70 mV, and spontaneous mEPSCs were recorded for five minutes. Amplitude and inter-event intervals (IEI) were analyzed with miniAnalysis (Synaptosoft), with an amplitude threshold of 3.5 pA.

#### Field potential recordings

For field potential experiments, pipettes were filled with 3 M NaCl and fEPSPs were measured at a stimulus intensity that elicited amplitudes that were ∼30–50% of the maximum. Input-output curves were generated by plotting the slope of fEPSPs as a function of the amplitude of the afferent fiber volleys (FV). To do so, neuronal responses were evoked by stimulating afferent fibers for 100 µs with current intensities ranging from 20 to 300 µA. LTP was elicited with a single train (1 s, 100 Hz). In LTD experiments, 17–21 day old mice were used. For the induction of LTD, a low-frequency stimulation was used, consisting of 900 pairs of stimuli (interval 50 ms) at 1 Hz. Paired pulse ratio (PPR) was analyzed by applying pairs of stimuli at the following inter-stimulus intervals (ISI; in ms): 10, 15, 25, 50, 75, 100, 150, and 200. Short-term depression was monitored during stimulation at 10 Hz for two full minutes in the presence of d-APV (Ascent Scientific).

### Behavior

Mice were housed in groups of three to five in standard Type II polycarbonate mouse cages at constant temperature (22±1°C) and humidity (50±5%) with food and water *ad libitum*. Experiments were performed during the light period. For all behavioral analyses, 8-week-old ADF-KO and CTR were used.

#### Contextual and cued fear conditioning

Experiments were performed as described previously [6]. Briefly, an operant chamber was used, equipped with tone stimulus, house light, and a stainless-steel rod floor through which footshocks could be administered, all controlled by Freezeframe software (Coulbourn Instruments). During habituation (Hab) of the training day, mice were placed individually and allowed to explore the chamber for 120 s. A continuous tone (3,600 Hz, 95 dB), serving as the conditioned stimulus (CS), was then presented for 30 s, that co-terminated with a footshock (0.5 mA for 2 s), which served as the unconditioned stimulus (US). Mice were removed from the chamber 30 s after CS/US and returned to their home cage. 24 hours later, they were re-exposed to the same chamber for 6 min to test contextual memory. Another 24 hours later, the auditory CS test was performed to test cued memory. Prior to this test, the chamber context was altered by covering the floor with a PVC base and the walls with colored plastic as well as by changing the background noise, the light conditions and the olfactory characteristics. During the first 3 min of the test, the CS was absent (pre-CS), after which it was turned on for 3 min. Fear responses during the contextual and CS tests were assessed by scoring the subjects' freezing response using FreezeView software (Coulbourn Instruments) complemented by the experimenter's scoring. Freezing was defined as the absence of any movement for at least 2 s, except breathing. Sometimes slight head movements and occasional tail rattling were observed.

*Two-choice serial reaction task* was performed essentially as described before [21]. During the experiment, mice had unlimited access to water, but food was restricted to 1.6 g per day. Experimental sessions were conducted in operant chambers (Med Associates) equipped with three nose-poke units on one wall, a wire grid floor, and a Plexiglas door. Operant chambers were located in sound and light-attenuated enclosures. The left and right nose-poke units had a light inside. After initial shaping to nose-poke for milk reward (10% condensed milk solution), mice were trained in a two-choice serial reaction time task. The stimulus light was presented randomly on the right or left nose-poke unit and mice were forced to respond by a nose-poke in the illuminated hole within the stimulus duration of 5 s in order to receive the milk reward. A nose-poke in the illuminated unit within the stimulus duration was recorded as a ‘correct response’ and rewarded with the house light on and the delivery of 0.02 ml of the milk reward for 5 s. Nose-pokes in the left or right unit that was not illuminated or no response within 5 s stimulus duration were recorded as ‘incorrect response’. A session was finished if the animal completed 50 correct responses or the run time exceeded 30 min.

### Statistical analysis

The unpaired two-tailed *t*-Student's test was used when comparing two sets of data with normal distribution. For the behavioral analysis, one-way ANOVA was used to assess statistical differences between genotypes.

## Results

### ADF is present at pre- and postsynaptic structures of excitatory synapses

**Figure 1:**
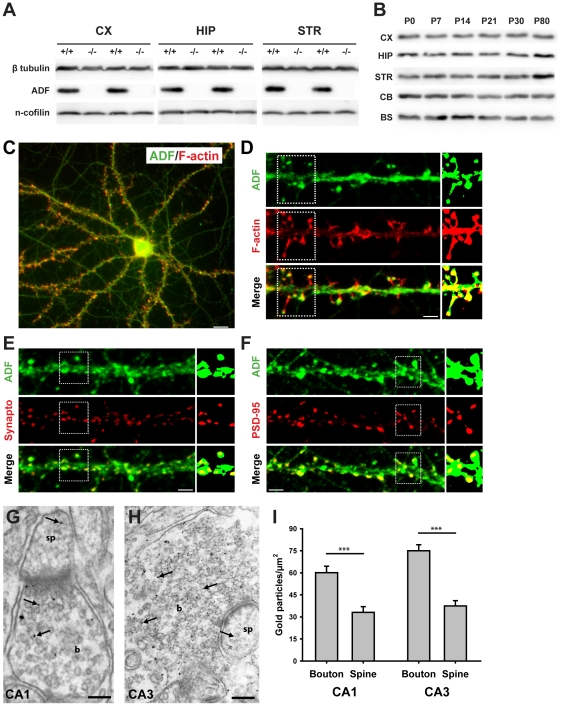
ADF is present in pre- and postsynaptic structures of excitatory synapses. (**A**) Immunoblot analyses demonstrated the specificity of the ADF antibody used for expression and localization studies: no band was detectable in cortical (CX), hippocampal (HIP) or striatal (STR) protein lysates from homozygous ADF mutants (−/−). N-cofilin levels were unchanged in lysates from homozygous ADF mutants. (**B**) Immunoblot analysis demonstrated the broad expression of ADF in the brain throughout postnatal development (postnatal day 0 (P0) to P80). CB: cerebellum, BS: brainstem. (**C**) Immunocytochemistry of cultured hippocampal pyramidal cells after 21 days in culture demonstrating the presence of ADF (green) in the cell body and in neurites. F-actin-rich structures were visualized with fluorescent-labeled phalloidin (red). Scale bar: 50 µm. (**D**) High magnification of a dendritc shaft, demonstrating the presence of ADF in phalloidin-positive, F-actin-rich structures such as dendritic spines. Scale bar: 5 µm. (**E**) Co-labeling with synaptophysin (synapto; red) revealed the presence of ADF (green) in presynaptic structures. Scale bar: 5 µm. (**F**) Likewise, ADF (green) co-localized with PSD-95 (red), a marker of the postsynaptic density. Scale bar: 5 µm. Small images in D–F: binary masks (green or red channel) and merged masks of dendritic structure indicated by dashed boxes. (**G+H**) Representative electron micrographs of the CA1 and CA3 *stratum radiatum* demonstrate localization of ADF in presynaptic boutons (b) and dendritic spines (sp). Arrows point to exemplarily gold particles that label ADF. Scale bars: 150 nm in G, 200 nm in H. (**I**) In the CA1 and CA3 region, density of gold particles was significantly higher in presynaptic boutons than in dendritic spines.

Previous studies reported the presence of ADF in the mouse brain [6], [16], [17], whereas its cellular and subcellular localization in the developing and adult brain has not yet been investigated in detail. Using a biochemical approach, we set out to comprehensively describe ADF expression in the mouse brain. Immunoblot analysis of protein lysates from ADF-deficient mice demonstrated the specificity of the ADF antibody used in this study (Fig. 1A). Notably, expression levels of n-cofilin were unchanged in protein lysates of the cerebral cortex (114.4±12.8% of control values, n = 4, P = 0.348), hippocampus (100.1±9.2%, n = 4, P = 0.997) or striatum (104.6±5.9%, n = 4, P = 0.702) from homozygous ADF mutants. We found the presence of ADF in cerebral cortex, hippocampus, striatum, cerebellum, and brainstem protein lysates throughout the first 80 days of postnatal development (Fig. 1B), demonstrating broad expression of ADF in the developing and adult mouse brain.

We next set out to examine the localization of ADF in excitatory neurons and investigated cultured hippocampal pyramidal cells. By immunocytochemistry, we found a homogenous distribution of ADF throughout the cytosol, including neurites (Fig. 1C). Using fluorescence-labeled phalloidin, we investigated co-localization of ADF with F-actin-rich structures, such as dendritic spines, and found ADF in the majority of dendritic spines independent of their shape (Fig. 1D, percentage of ADF-positive spines: mushroom-like: 95.5%, stubby: 84.4%, filopodia: 78.9%; numbers of spines analyzed: mushroom-like: 110, stubby: 78, filopodia: 102). Co-localization experiments, employing synaptophysin and PSD-95 as synaptic markers, demonstrated ADF in 82.5% of synaptophysin-positive presynaptic terminals and in 89.7% of PSD-95-positive postsynaptic structures (Fig. 1E–F; numbers of structures analyzed: synaptophysin: 78, PSD-95: 64).

Immuno-electron microscopy of hippocampal CA1 and CA3 *stratum radiatum* confirmed the synaptic localization of ADF (Fig. 1G–H). We found all presynaptic terminals (n = 51 for CA1, n = 19 for CA3) being labeled with gold particles and a substantial fraction of postsynaptic dendritic spines, too (percentage of ADF-positive spines: CA1: 79.5%, n = 57; CA3: 94.3%, n = 35). Our analyses revealed a higher gold particle density in presynaptic terminals when compared to postsynaptic dendritic spines (Fig. 1I; CA1: 60.1±4.4 gold particles/µm^2^ in presynaptic terminals; 33.1±3.9 gold particles/µm^2^ in spines; *P*<0.001; CA3: 75.0±3.9 gold particles/µm^2^ in presynaptic terminals; 37.5±3.5 gold particles/µm^2^ in spines; *P*<0.001). Taken together, our data demonstrate the presence of ADF in pre- and postsynaptic structures of excitatory synapses, with ADF being particularly enriched in presynaptic terminals.

### Unaltered synapse density and morphology in CA1 pyramidal cells of ADF mutants

**Figure 2:**
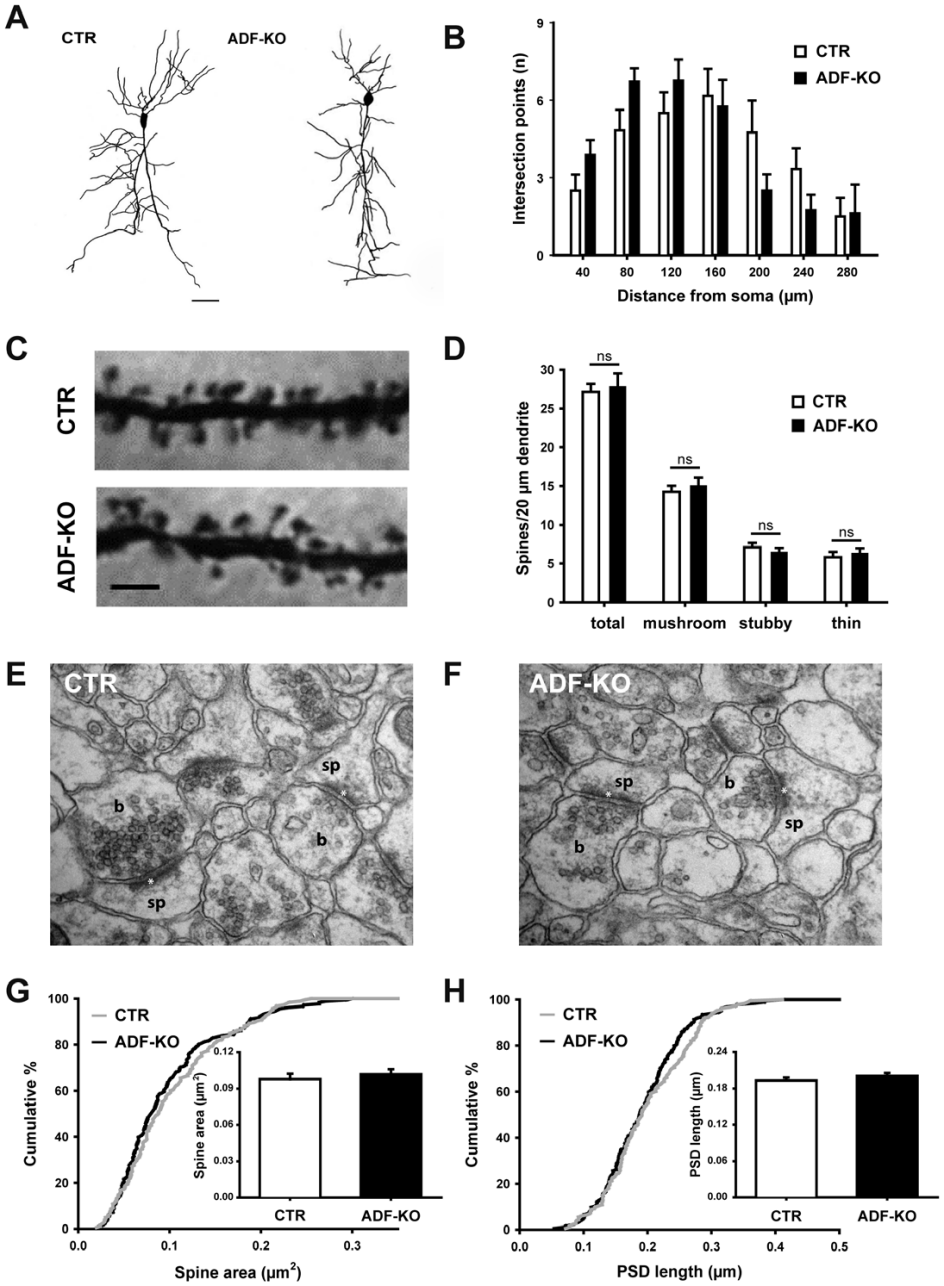
Unaltered dendritic spine density and morphology in CA1 pyramidal cells. (**A**) Representative examples of Golgi-stained CA1 pyramidal cells indicate similar branching and complexity of the dendritic trees in controls (CTR) and ADF mutants (ADF-KO). Scale bar: 50 µm. (**B**) Sholl analysis of the apical dendritic tree in the *stratum radiatum* revealed no difference between both genotypes (n = 8 for CTR; n = 6 for ADF-KO). (**C**) Representative images of 2^nd^ order dendritic branches from Golgi-stained pyramidal cells in the *stratum radiatum* of CA1. Scale bar: 2.5 µm. (**D**) The total number of spines (CTR: 27.1±0.2 spines/20 µm, ADF-KO: 27.9±0.5 spines/20 µm, P = 0.715) as well as the numbers of mushroom-like (CTR: 14.2±0.2 spines/20 µm, ADF-KO: 15.0±0.2 spines/20 µm, P = 0.540), stubby (CTR: 7.1±0.1 spines/20 µm, ADF-KO: 6.5±0.1 spines/20 µm, P = 0.435) and thin spines were unaltered in ADF-KO (CTR: 5.8±0.1 spines/20 µm, ADF-KO: 6.3±0.1 spines/20 µm, P = 0.583; more than 1,000 µm dendritic length was analyzed in four mice per genotype). (**E+F**) Representative electron micrographs of CA1 *stratum radiatum* from a CTR and an ADF-KO mouse. Image size: 2.27 µm^2^; b: presynaptic bouton, sp: postsynaptic spine, *: postsynaptic density. (**G**) Spine area (CTR: 0.098±0.004 µm^2^, n = 181 spines from 3 mice; ADF-KO: 0.102±0.004 µm^2^, n = 185/3; P = 0.512) and (**H**) PSD length (CTR: 0.193±0.005 µm, n = 184 spines from 3 mice; ADF-KO: 0.201±0.005 µm, n = 183/3; P = 0.277) were unchanged in ADF-KO as deduced from the cumulative distribution curves and the mean values (insets).

In order to assess the relevance of ADF for synapse structure and function, we analyzed a recently generated mouse model with a targeted inactivation of ADF (ADF-KO) [17]. To verify whether deletion of ADF affects neuronal morphology or dendritic spine density, we investigated Golgi-stained CA1 hippocampal pyramidal cells (Fig. 2A). By means of a Sholl analysis of the dendritic trees in CA1 *stratum radiatum*, we revealed normal branching and complexity of pyramidal cell dendrites in ADF-KO (Fig. 2B). The same result was obtained in *stratum oriens* (data not shown).

Actin is essential for the morphology of dendritic spines [4], and dysregulation of the postsynaptic actin cytoskeleton can disturb dendritic spine density and morphology [6], [15]. To prove whether ADF is relevant for spine density and morphology, we quantified the spine numbers of 2^nd^ order dendritic branches in CA1 *stratum radiatum* (Fig. 2C). We found similar total spine numbers in controls (CTR) and ADF-KO neurons (CTR: 26.6±0.9 spines/20 µm dendrite, n = 33 cells from three mice; ADF-KO: 27.9±1.2 spines/20 µm dendrite, n = 31/3; P = 0.382). Moreover, by comparing both groups, we found no differences in the density of mushroom-like, stubby, or thin spines (Fig. 2D) or in the dendritic spine length (CTR: 0.946±0.018 µm, ADF-KO: 0.930±0.019 µm, n≥259 from three mice and at least three CA1 pyramidal cells from each mouse, P = 0.539).

In accordance with normal spine numbers, the density of excitatory synapses in CA1 *stratum radiatum* was unaltered in ADF-KO as revealed by analyzing electron micrographs (Fig. 2E–F; CTR: 0.555±0.016 synapses/µm^2^, n = 145 micrographs from three mice; ADF-KO: 0.596±0.016 synapses/µm^2^, n = 145/3; P = 0.069). Moreover, synapse ultrastructure appeared to be independent of ADF activity as spine area (Fig. 2G), PSD length (Fig. 2H), and presynaptic bouton size (data not shown) were unchanged in ADF-KO. Additionally, we found similar densities of synaptic vesicles (CTR: 109.2±5.6 vesicles/µm^2^, n = 70 boutons from three mice; ADF-KO: 124.6±7.9 vesicles/µm^2^, n = 89/3; P = 0.113) and similar numbers of docked vesicles (CTR: 11.3±0.8 vesicles/µm, n = 70 active zones from three mice; ADF-KO: 11.6±0.7 vesicles/µm, n = 89/3; P = 0.776) in electron micrographs from CTR and ADF-KO. Taken together, our data revealed that ADF is not crucial for the density, morphology and ultrastructure of excitatory synapses. In addition, organization of synaptic vesicles is independent of ADF.

### ADF is dispensable for synapse physiology

**Figure 3:**
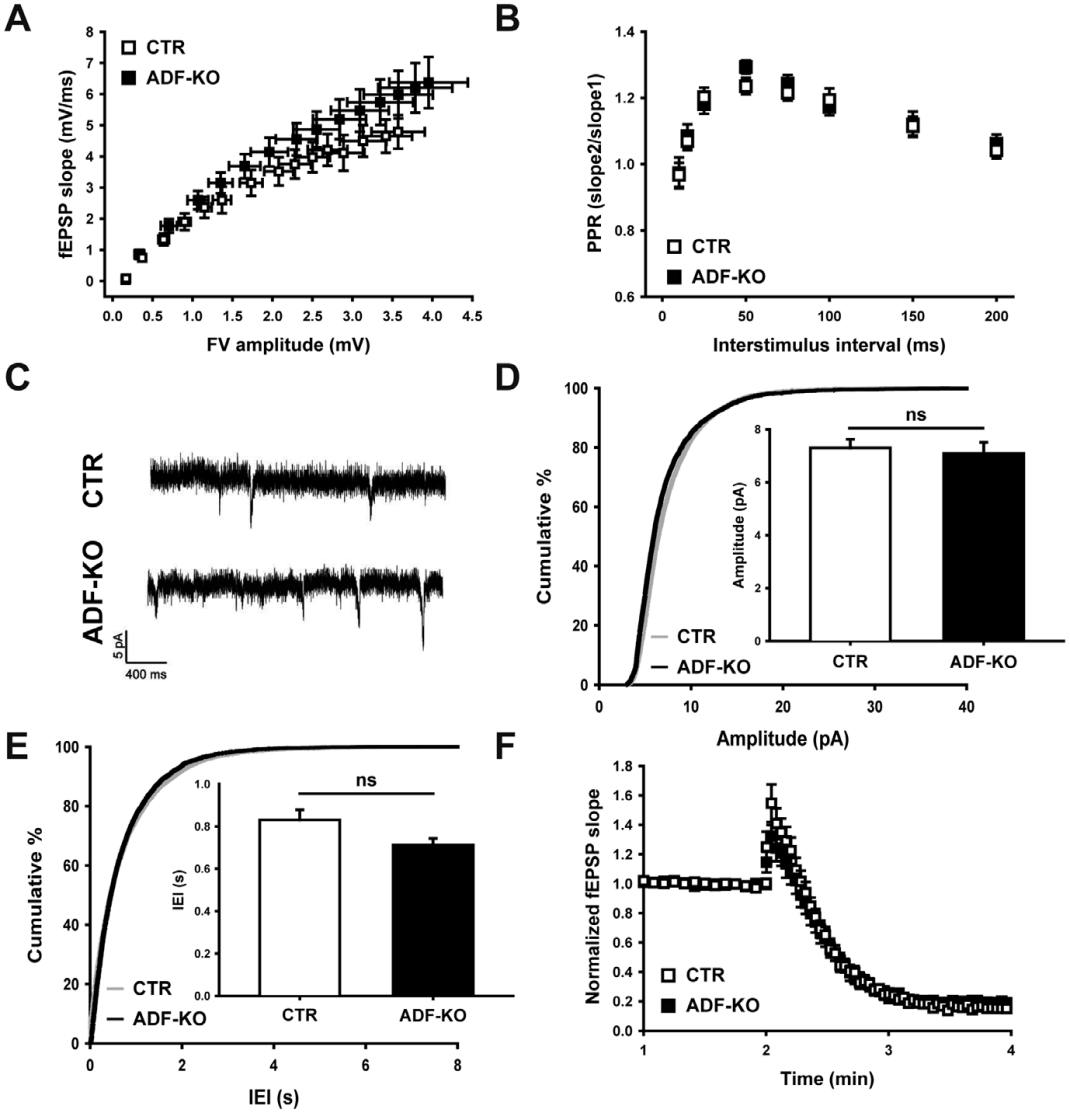
Basal synaptic transmission and presynaptic physiology were independent of ADF. (**A**) Input-output curves were not different between CTR and ADF-KO (n = 12 for CTR; n = 16 for ADF-KO), indicating unchanged basal synaptic transmission in ADF-KO. (**B**) Paired pulse ratio (PPR) at different interstimulus intervals (10 to 200 ms) was unaltered in ADF-KO (n = 12 for CTR; n = 18 for ADF-KO). (**C**) Exemplary traces showing miniature excitatory postsynaptic currents (mEPSC) recorded from a CTR (upper trace) and an ADF-KO pyramidal cell (lower trace). (**D+E**) Both mEPSC amplitudes and interevent intervals (IEI) were unaltered in ADF-KO as deduced from the cumulative curves and mean values (insets; mEPSC amplitudes: CTR: 7.29±0.33 pA, n = 14 cells from 7 mice; ADF-KO: 7.09±0.42, n = 9/3; P = 0.699; IEI: CTR: 0.831±0.049 s; ADF-KO: 0.712±0.031 s; P = 0.089) were unaltered in ADF-KO. (**F**) Additionally, presynaptic short-term vesicle depression induced by 10 Hz stimulation for 2 min was similar in CTR and ADF-KO.

N-cofilin is required for postsynaptic plasticity, but not essential for presynaptic function [6]. To assess the scenario with ADF, we next set out to test whether ADF plays a similar or complementary role in synapse physiology. We first investigated whether ADF is relevant for basal synaptic transmission. We stimulated the Schaffer collateral pathway with current amplitudes ranging from 20 to 300 µA and recorded afferent fiber volleys (FV) and excitatory postsynaptic field potentials (fEPSP) in the CA1 region of acute hippocampal slices. The resulting input-output curves showed equal presynaptic FV amplitudes and fEPSP slopes in CTR and ADF-KO slices (Fig. 3A). Likewise, no differences in the paired-pulse ratio (PPR) were found between CTR and ADF-KO slices at any of the investigated inter-stimulus intervals (10–200 ms; Fig. 3B). Moreover, neither the amplitude (Fig. 3C–D) nor the inter-event-interval (Fig. 3E) of miniature excitatory postsynaptic currents (mEPSC) was altered in patch-clamped CA1 pyramidal cells from ADF-KO. We also depleted the presynaptic vesicle pool with a short-term depression protocol (10 Hz stimulation for 2 min, see [6]), which again showed no difference between CTR and ADF-KO preparations (Fig. 3F). In summary, based on our data, we exclude a crucially important contribution of ADF for basal synaptic physiology, synaptic vesicle recruitment, and exocytosis or short-term plasticity.

**Figure 4:**
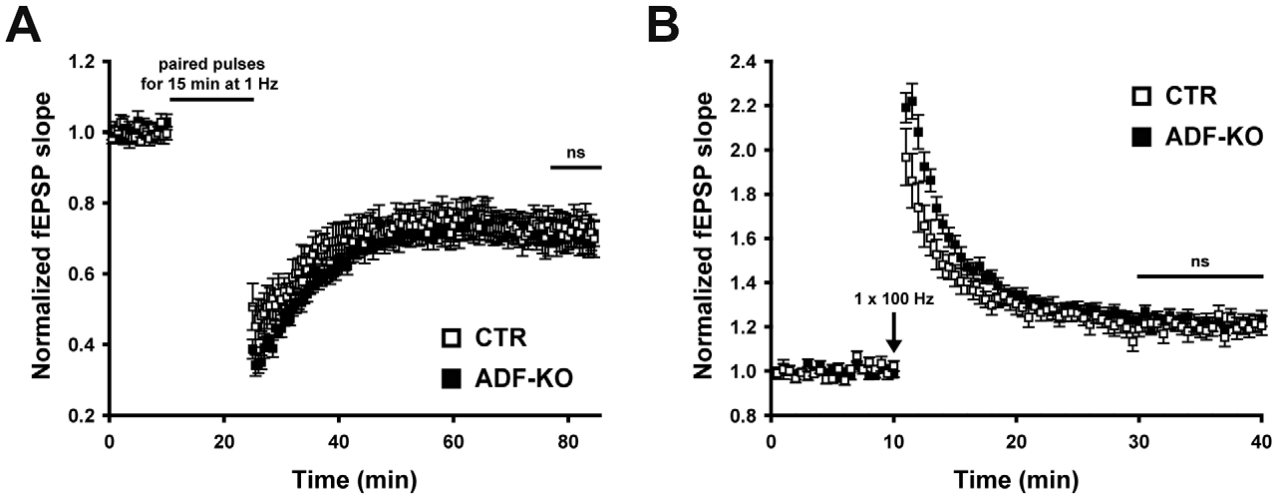
ADF is dispensable for synaptic plasticity. (**A**) Long-term depression induced by low frequency stimulation (1 Hz for 15 min) with paired pulses (interpulse interval 50 ms) showed no difference between CTR and ADF-KO (last 10 min of recording; CTR: 0.725±0.042, n = 8; ADF-KO: 0.706±0.038, n = 9; P = 0.739). (**B**) Likewise, long-term potentiation induced by a single tetanic stimulation with 100 Hz for 1 s was indistinguishable between CTR and ADF-KO (last 10 min of recording; CTR: 1.205±0.035, n = 12; ADF-KO: 1.221±0.028, n = 12; P = 0.732).

As it turned out that ADF is not relevant for presynaptic activity, we next investigated whether ADF has any importance for postsynaptic plasticity. We first tested whether long-term depression (LTD) depends on ADF activity as we previously found impaired LTD in n-cofilin mutants [6]. To do so, we induced LTD by low frequency paired stimulation for 15 min with 1 Hz. We found similar responses in CTR and ADF-KO slices (Fig. 4A). Likewise, long-term potentiation (LTP) induced by a single tetanic stimulation of 100 Hz for 1 s was independent of ADF activity (Fig. 4B). In summary, our electrophysiological analysis revealed that pre- and postsynaptic mechanisms are not affected in the absence of ADF.

### Normal learning and memory in ADF mutants

**Figure 5:**
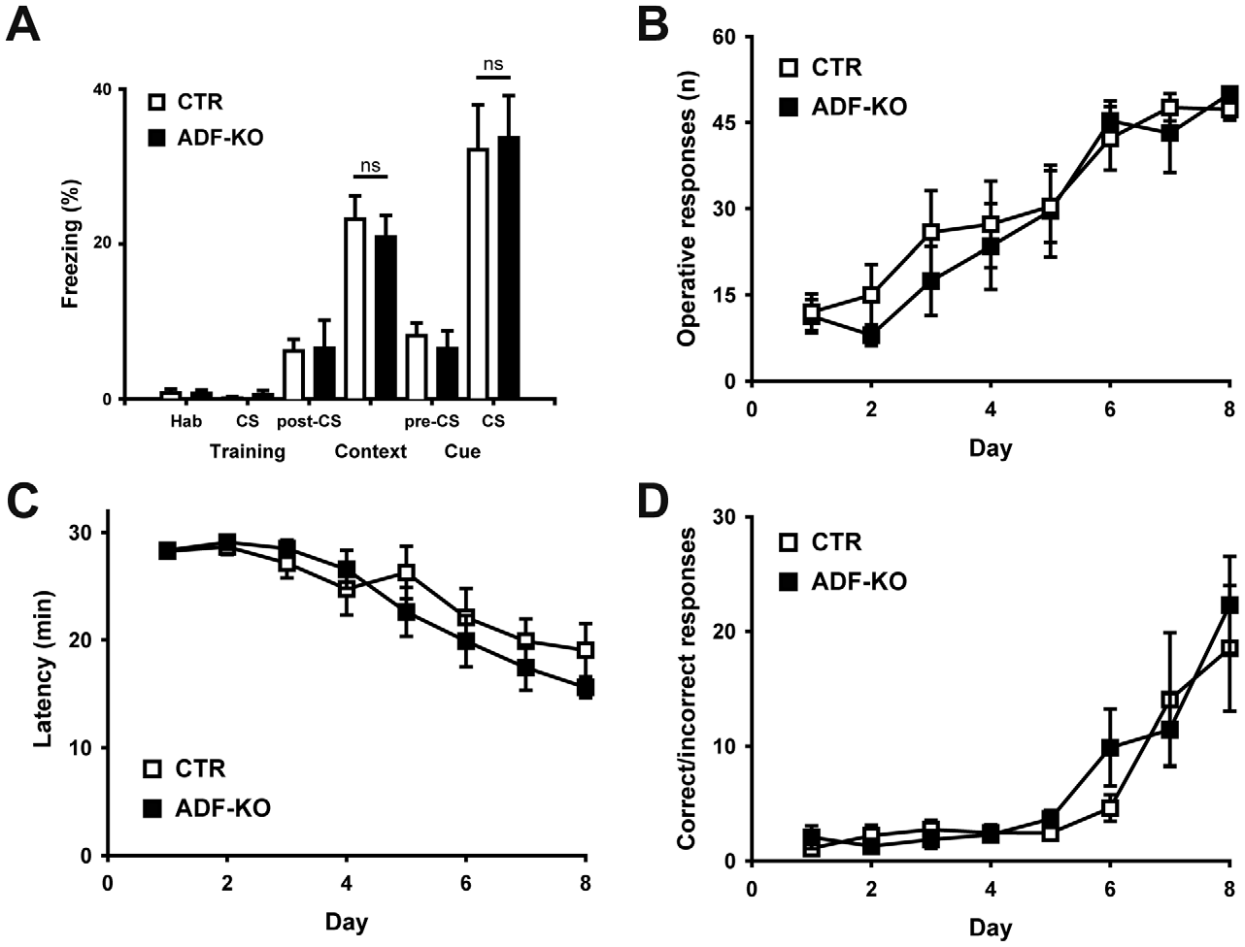
Normal learning and memory in ADF-KO. (**A**) Freezing behavior in a fear-conditioning paradigm during the context (CTR: 23.4±3.0%, n = 17; ADF-KO: 21.0±2.8, n = 15; P = 0.577) and cue testing (CTR: 32.3±5.8%; ADF-KO: 33.8±5.4; P = 0.848) was unchanged in ADF-KO. HAB: habituation, CS: conditioned stimulus. (**B–D**) Likewise, a two-choice serial reaction task revealed no learning defect in ADF-KO. (**B**) The number of operative responses as well as (**C**) the latency for reaching the maximum of 50 operative responses or (**D**) the ratio of correct versus incorrect responses were unchanged between CTR and ADF-KO (n = 8 for both genotypes).

Defective learning was recently described for n-cofilin mutants [6]. Specifically, these mutants performed weaker in the Morris water maze (MWM), but also in a contextual and cued fear conditioning task (CFC). We therefore wanted to assess whether learning is affected in ADF-KO, too. As ADF-KO display a mild corneal hypoplasia [17], [22] and performed significantly weaker in the visible platform testing of the MWM (data not shown), we excluded the MWM paradigm from our behavioral analysis and instead examined memory formation in ADF-KO by performing CFC. Mice of both groups showed virtually no freezing during the two minutes of habituation and the 30 s of cue stimulation (tone) that was coupled to an aversive experience (footshock). In the post-cue period, both groups showed similar freezing rates (Fig. 5A; CTR: 6.4±1.5%, n = 17; ADF-KO: 6.7±3.6%, n = 15; P = 0.917). When testing contextual memory 24 h after training, CTR and ADF-KO displayed significantly higher freezing rates compared to habituation of the training day (CTR: habituation: 0.9±0.5%, context: 23.4±3.0%; P<0.001; ADF-KO: habituation: 0.9±0.5%, context: 21.0±2.8%; P<0.001). Likewise, when testing cued memory 48 h after training, CTR (habituation: 8.3±1.7%, cue: 32.3±5.8%; P<0.001) and ADF-KO (habituation: 6.7±2.3%, cue: 33.8±5.4%; P<0.001) showed higher freezing rates when compared to the habituation of the cue experiment. However, neither in the context nor in the cue experiment did we find significant differences in freezing rates between CTR and ADF-KO (context: P = 0.577; cue: P = 0.848). Thus, our data demonstrate that the inactivation of ADF does not appear to affect associative memory formation in mice.

In a second approach, we challenged ADF-KO in a two-choice serial reaction task in which food-deprived mice were trained for nose-poking in response to a light stimulus for receiving a milk reward [21]. In this experiment, ADF-KO behaved similarly to CTR, because the number of operative responses (right nose-poking unit and reward receipt; Fig. 5B) and the latency for reaching the maximum of 50 operative responses (Fig. 5C) did not differ from that of CTR. In addition, reference memory, as deduced from the ratio of correct versus incorrect responses, did not differ between CTR and ADF-KO (Fig. 5D). Taken together, our data show that both learning and memory are unaffected in the absence of ADF.

### N-cofilin is capable of compensating the loss of ADF at synapses

**Figure 6:**
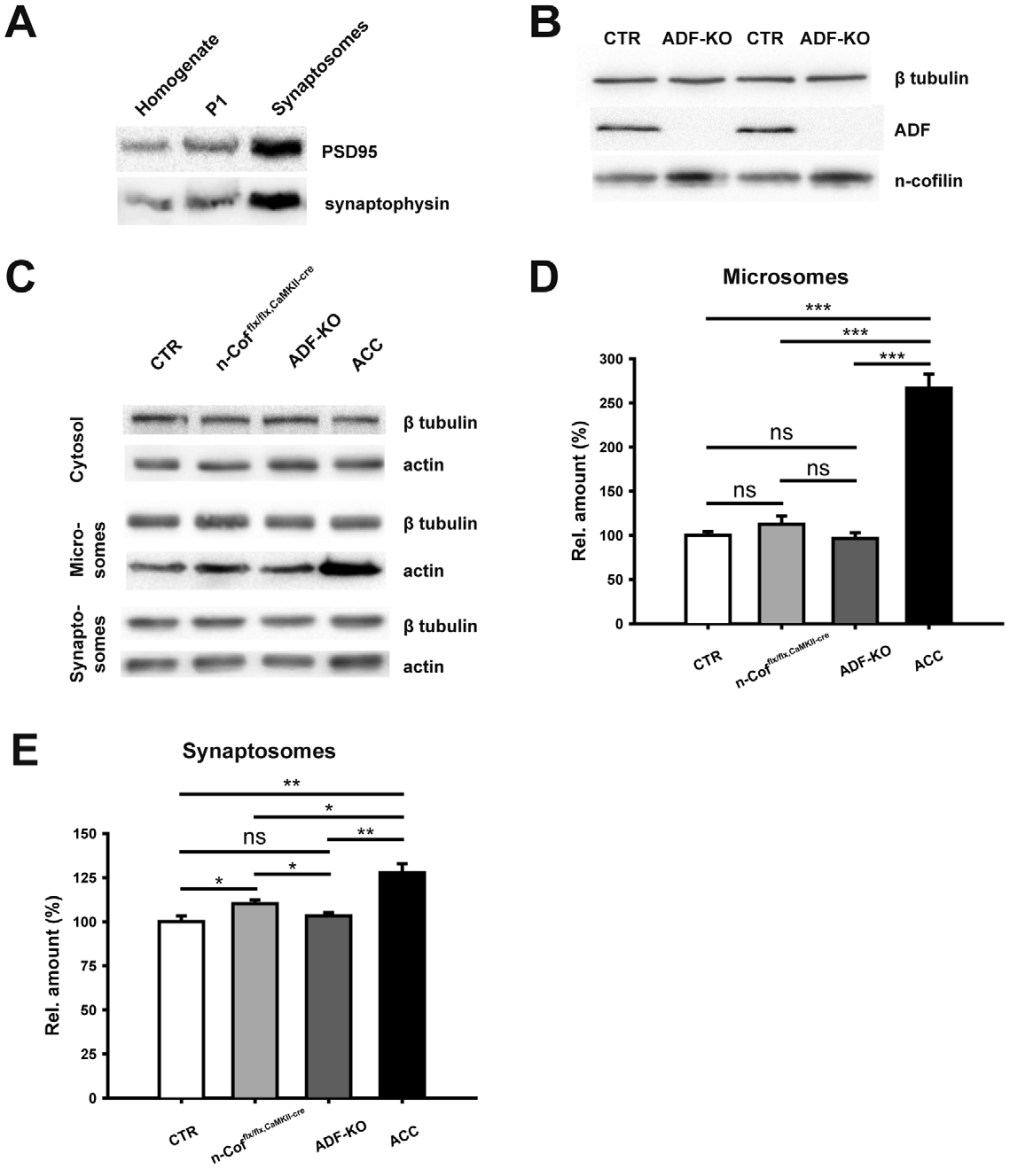
N-cofilin compensates the loss of ADF at synapses. (**A**) Immunoblot analyses show enrichment of synaptic markers (PSD-95, synaptophysin) in synaptosomes. Equal protein load was verified by Coomassie staining of SDS-PAGEs. Compared to total protein lysates (homogenate) and the organelle- and nuclei-containing fraction P1 PSD-95 and synaptophysin signals were increased in synaptosomes. (**B**) Immunoblots showing that synaptic n-cofilin levels were increased in synaptosomes from ADF-KO. (**C**) Immunoblot analysis demonstrating equal cytosolic actin levels in CTR, n-cofilin mutants (n-Cof^flx/flx,CaMKII-cre^), ADF-KO and double mutants (ACC) that lack both ADF and n-cofilin. Conversely, actin levels were increased in microsomal and synaptosomal preparations from ACC mice. (**D**) Quantification of microsomal actin levels. In microsomes from ACC mice, actin levels were significantly increased to 266.8±15.8% of CTR levels (n = 4; P<0.001) and significantly higher compared to n-Cof^flx/flx,CaMKII-cre^ mice (P<0.001) or ADF-KO (P<0.001). No significant increase in actin levels was found in microsomes from ADF-KO or n-cofilin mutants. (**E**) Quantification of actin levels in synaptosomes. Actin levels in synaptosomes were significantly increased in n-cofilin mutants (+10.1±2.1%; n = 4; P<0.05) and ACC mice (+27.8±5.2%; n = 4; P<0.01). In ACC mice, synaptosomal actin levels were significantly higher than in synaptosomes from n-cofilin mutants (P<0.05) or ADF-KO (P<0.01). No increase was found in synaptosomes from ADF-KO.

The lack of any observable synaptic defect in ADF-KO led us to speculate that ADF inactivation might be functionally rescued. We therefore investigated synaptic levels of n-cofilin in ADF-KO, another member of the ADF/cofilin family that is broadly expressed in the brain and located in excitatory synapses [17], [23]. We prepared hippocampal synaptosomes from CTR and ADF-KO. Immunoblot analyses demonstrated the enrichment of synaptic proteins in such synaptosomal fractions (Fig. 6A). By comparing CTR synaptosomes with those from mutants, we found significantly increased n-cofilin levels in synaptic structures from ADF-KO (Fig. 6B; +42.6±9.8%; n = 6; P = 0.005). Thus, our data suggest that n-cofilin can compensate for the loss of ADF in synapses. In order to prove our suggestion, we employed compound mutants with a systemic inactivation of ADF and a deletion of n-cofilin in principal neurons of the postnatal forebrain (ADF^−/−^/n-Cof^flx/flx,CaMKII-cre^ mice, in the following called ACC mice). Immunoblot analysis revealed normal actin levels in cytosolic fractions from hippocampal preparations in all four genotypes tested (Fig. 6C). Conversely, compared to CTR actin levels in ACC mice were strongly increased in membrane protein-enriched fractions that also contained actin (microsomes; +166.8±15.8%, P<0.001) [18], but not in the microsomes obtained from ADF-KO (−3.4±6.4%, P = 0.673) or n-cofilin mutants (n-Cof^flx/flx,CaMKII-cre^ mice; +12.8±9.1%, P = 0.251; n = four for all genoytpes; Fig. 6D). Additionally, we found significantly more actin in synaptosomes from ACC mice than in CTR or single mutants (Fig. 6C,E; ADF-KO: +3.2±2.0%, n-cofilin mutants: +10.1±2.1%, ACC: +27.8±5.2%; n = 4 for all genotypes; ACC vs. CTR: P = 0.004, ACC vs. ADF-KO: P = 0.004, ACC vs. n-cofilin mutants: P = 0.019). Together, these data demonstrate changes in actin regulation induced by the compound inactivation of ADF and n-cofilin. They show that n-cofilin has the capacity to compensate for the loss of ADF at synapses and therefore explain the lack of any observable synaptic defect in ADF-KO.

## Discussion

This study focused on the distribution and function of the F-actin depolymerizing protein ADF in excitatory synapses. Our analysis was inspired by previous reports which had demonstrated an important role of F-actin depolymerizing activity in synapse physiology, learning, and memory [6], [7], [11], [12], [15]. By immunocytochemistry and immuno-EM, we found that ADF is present in virtually all excitatory synapses of hippocampal pyramidal cells and located in presynaptic as well as postsynaptic structures. As previously described for outer hair cell-innervating medio-olivocochlear projections [24], we found an enrichment of ADF in presynaptic terminals of hippocampal pyramidal cells, whereas the other ADF/cofilin family member, n-cofilin, reportedly is enriched in postsynaptic spines [23]. The complementary distribution pattern of ADF and n-cofilin implies that these two molecules fulfill diverse functions in excitatory synapses. In fact, n-cofilin was shown to be critical for dendritic spine morphology and postsynaptic plasticity, but not for presynaptic physiology [6]. As inactivation of LIMK1, a negative regulator of both ADF and n-cofilin, impairs postsynaptic plasticity but also results in defective presynaptic physiology [15], we speculated that ADF may have an important role in presynaptic mechanisms. Surprisingly however, presynaptic physiology was fully preserved in ADF-KO. In addition, we found that neuronal complexity, synapse morphology, LTD, LTP as well as learning and memory do not require ADF. Taken together, our study shows that ADF inactivation does not interfere with neuronal differentiation and synaptic function. By contrast, neuronal complexity, brain development, and synaptic function are severely impaired in n-cofilin mutants [6], [17]. We therefore conclude that n-cofilin is the major ADF/cofilin isoform in the brain - a finding that is consistent with the fact that the amount of n-cofilin in the brain is six to ten times higher than that of ADF [17], [25].

The lack of any synaptic defect in ADF-KO led us to speculate that n-cofilin has the capacity to compensate for the loss of ADF. In line with this hypothesis, we found increased n-cofilin levels in synaptic structures, but not in hippocampal total protein lysates from ADF-KO. Likewise, ADF levels were elevated in synaptic structures of n-cofilin mutants [6], yet unchanged in total brain lysates [17]. Together, these findings imply functional redundancy of ADF and n-cofilin specifically in synaptic compartments.

We found increased actin levels in microsomal preparations from double mutants lacking both ADF and n-cofilin. Increased microsomal actin content likely reflects increased F-actin levels and is consistent with the loss of F-actin depolymerizing activity in these mutants. As microsomal actin levels were unchanged in preparations from ADF or n-cofilin mutants, our data directly prove compensatory effects in single mutant mice. Additionally, synaptic actin levels were higher in double mutants than in ADF or n-cofilin single mutants. Therefore, we conclude that ADF and n-cofilin have the capacity to compensate each other in synaptic structures. The lack of any synaptic defect in ADF-KO implies considerable compensation of the ADF inactivation by n-cofilin. Conversely, ADF, presumably because of its predominantly presynaptic location, fails to countervail the loss of n-cofilin in postsynaptic structures [6]. Further analysis of double mutant mice (ACC mice) is needed for a comprehensive understanding of ADF and n-cofilin function in synapse physiology and is likely to ultimately unravel whether, and to what degree, ADF and n-cofilin are relevant for presynaptic physiology.

In summary, our data demonstrate a pre- and postsynaptic localization of ADF in excitatory synapses and the enrichment of ADF in presynaptic terminals. By analyzing ADF mutant mice, we show that ADF inactivation has no adverse effects on neuron morphology, synapse ultrastructure, synaptic physiology, or learning and memory, likely due to compensation by n-cofilin. Our results demonstrate that ADF and n-cofilin cooperate in synapses where they are relevant for controlling actin levels. Future analysis of double mutants and the comparison with the single knockout may help to dissect the synaptic function of ADF.
